# Blind nasoenteric tube placement risks: A case report of misplacement into the pleural cavity

**DOI:** 10.1097/MD.0000000000041101

**Published:** 2025-01-10

**Authors:** You Yuan, Wei Zhong, Huiming Gao, Xia Zhang, Junxi Chen, Yuanqin Qing, Rujun Hu

**Affiliations:** a Department of Critical Care Medicine, Affiliated Hospital of Zunyi Medical University, Zunyi, Guizhou, People’s Republic of China; b Department of Nursing, Affiliated Hospital of Zunyi Medical University, Zunyi, Guizhou, People’s Republic of China; c School of Nursing, Zunyi Medical University, Zunyi, Guizhou, People’s Republic of China.

**Keywords:** case report, enteral nutrition, nasoenteric tube, patient safety, pleural cavity

## Abstract

**Rationale:**

Enteral nutrition is a critical component of care for critically ill patients. However, the blind insertion of a nasoenteric tube, despite being a simple procedure, carries inherent risks that necessitate a reevaluation of the technique.

**Patient concerns:**

A case of a 60-year-old female experienced the rare yet critical complication of a misplaced nasoenteric tube entering the thoracic cavity during a blind insertion procedure for enteral nutrition following a liver transplant.

**Diagnosis:**

Following liver transplantation, the patient was diagnosed with severe pneumonia, a right-sided hydropneumothorax, and severe malnutrition.

**Interventions:**

After the misplacement of the nasoenteric tube into the pleural cavity was detected from the chest X-ray, the tube was immediately removed, and the pneumothorax was actively managed. Subsequently, with the support of contrast radiography, the nasoenteric tube was successfully reinserted to provide the patient with nutritional support and promote rehabilitation.

**Outcomes:**

The patient responded well to the intervention and was discharged in stable condition following complete recovery.

**Lessons:**

The case prompts a reevaluation of blind placement techniques and calls for the adoption of more reliable technologies to prevent similar incidents and ensure patient safety, such as electromagnetic and visualized guided placement technique.

## 1. Introduction

Enteral nutrition support is essential for maintaining intestinal function and immune function, promoting rapid recovery, and improving patient prognosis. In economically underdeveloped regions of Western China, blind nasoenteric tube placement is common due to its simplicity. However, this method carries significant risks, as illustrated by a rare but severe case of pneumothorax resulting from such a placement. This report provides detailed imaging and records from the procedure, offering a comprehensive analysis of the risks involved. The case prompts a reevaluation of blind placement techniques and calls for the adoption of more reliable technologies to prevent similar incidents and ensure patient safety.

## 2. Patient information

**Figure 1.**
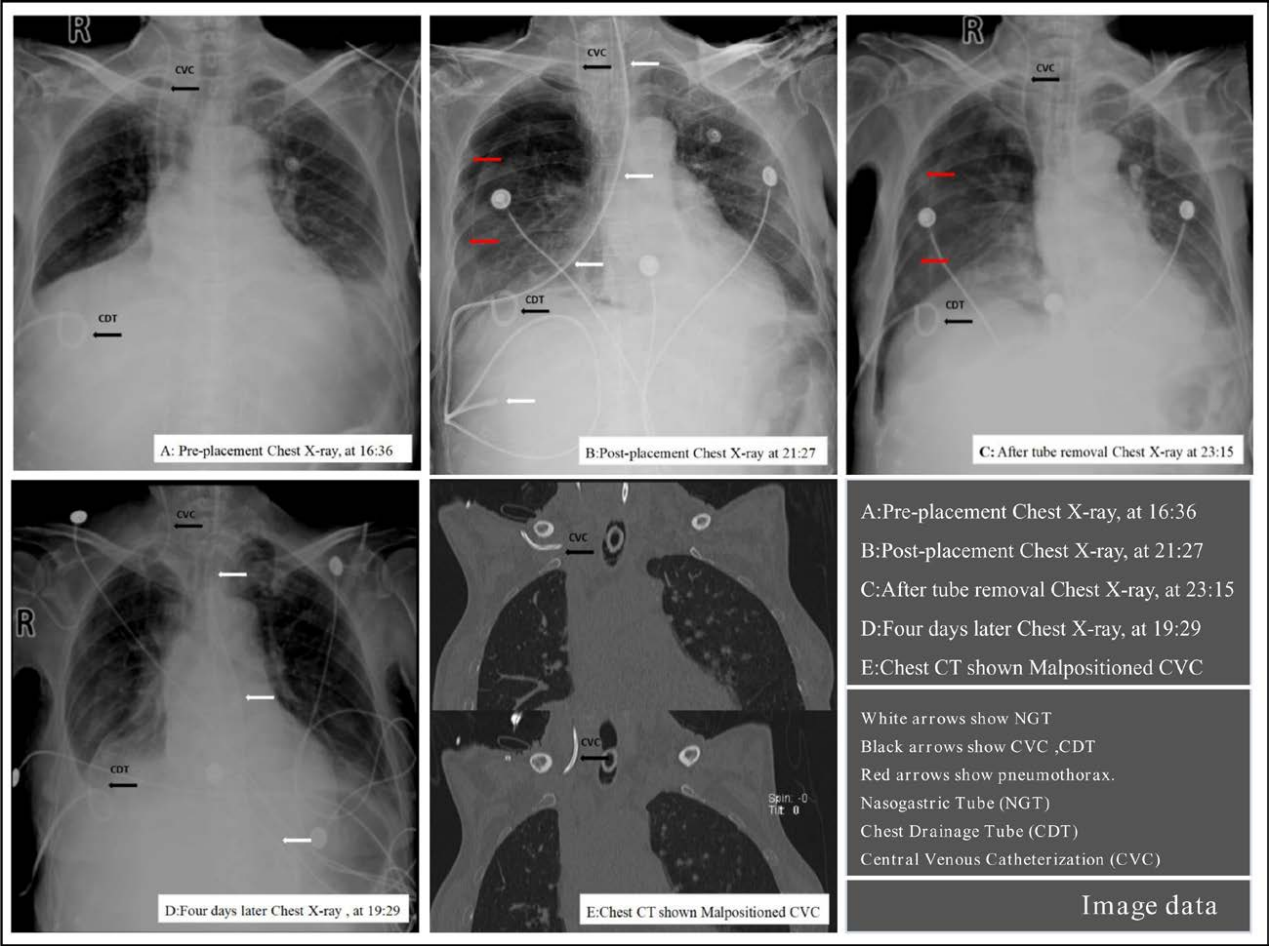
Image data.

The patient, a 60-year-old woman (weight: 48 kg, height: 162 cm, BMI: 18.29), underwent orthotopic liver transplantation 19 days earlier for a giant hepatic hemangioma. Her postoperative recovery was poor, requiring tracheal intubation and mechanical ventilation. She had an indwelling right internal jugular vein catheter, a right chest drainage tube, and a T-tube for bile drainage. The patient was only receiving continuous analgesia with Remifentanil at 1.7 μg/kg/h, had a Richmond agitation-sedation scale score of 0, and a critical-care pain observation tool score of 0. She had lost weight of more than 15 kg in the past 3 months and her nutritional risk screening 2002 (NRS 2002) score was 6. Her medical history included pulmonary encephalopathy, diaphragmatic injury, and a right-sided pneumothorax, along with a previous gastric tube placement. There were no notable family or psychosocial issues, nor relevant genetic information. The current medical order was to place a nasoenteric tube for enteral nutrition. preplacement chest X-ray was taken prior to the procedure at 16:36 (Fig. 1A) showed no obvious pneumothorax.

## 3. Timeline: tube placement process

The nasoenteric tube placement began at 19:50, performed by a certified critical-care nurse with 13 years of experience and assisted by a colleague with 4 years of experience. Prior to the procedure, all necessary supplies were prepared, and the patient received 10 mg of intravenous metoclopramide to enhance gastrointestinal motility. During the blind placement, the nasoenteric tube passed through the nasopharynx without resistance. At a depth of 15 to 20 cm, the patient showed no signs of choking or discomfort, and vital signs were stable (SpO_2_ 99%). The main operator continued to advance the tube to a depth of 45 cm. At this point, by the method of aspiration, the main operator, upon seeing the yellow liquid drawn up into the syringe, made a preliminary conclusion (incorrectly) that the tube had reached the stomach. Upon auscultation over the stomach area, the assistant heard the loud gurgling sounds, which led them to further confirm (mistakenly) that the tip of the tube was located within the stomach. Furthermore, the operator submerged the tube’s end in water for a bubble test, with no bubbles observed, which once again confirmed (inaccurately) that the tube had not entered the trachea but was within the stomach. Throughout the procedure, the patient did not exhibit choking or discomfort, and vital signs remained normal (blood pressure 127/65 mm Hg, heart rate 80 bpm, respiratory rate 18/min, SpO_2_ 99%). Following that the patient was positioned on her right side, and the main operator proceeded to advance the nasoenteric tube to a depth of 95 cm, the blind placement of the nasoenteric tube was completed in 15 minutes. Immediately afterward, a radiology technician was contacted to perform a bedside X-ray to confirm the position of the nasoenteric tube. However, the post-placement chest x-ray at 21:27 (Fig. 1B) indicated a right pneumothorax with approximately 30% lung compression. The nasoenteric tube was urgently removed at 22:40. The removal process was smooth, with the tube found intact. A follow-up bedside chest X-ray at 23:15 (Fig. 1C) showed worsening pneumothorax, with 40% to 45% lung compression. Despite the complications, the patient’s vital signs remained stable throughout the procedure and the subsequent intervention.

## 4. Follow-up and outcomes

Two days after the initial complication, the nasoenteric tube was successfully reinserted under fluoroscopic guidance by the interventional team. A chest X-ray performed 4 days later at 19:29 (Fig. 1D) confirmed resolution of the pneumothorax and repositioning of the central venous catheter. Further imaging with chest CT (Fig. 1E) provided detailed confirmation of these findings. With the reestablishment of enteral nutrition, the patient’s clinical condition stabilized and showed marked improvement. She was discharged from the ICU after 12 days of supportive care and continued to recover in the general ward. One month after the event, the patient was discharged from the hospital in good condition.

## 5. Root cause analysis results and measures

**Figure 2.**
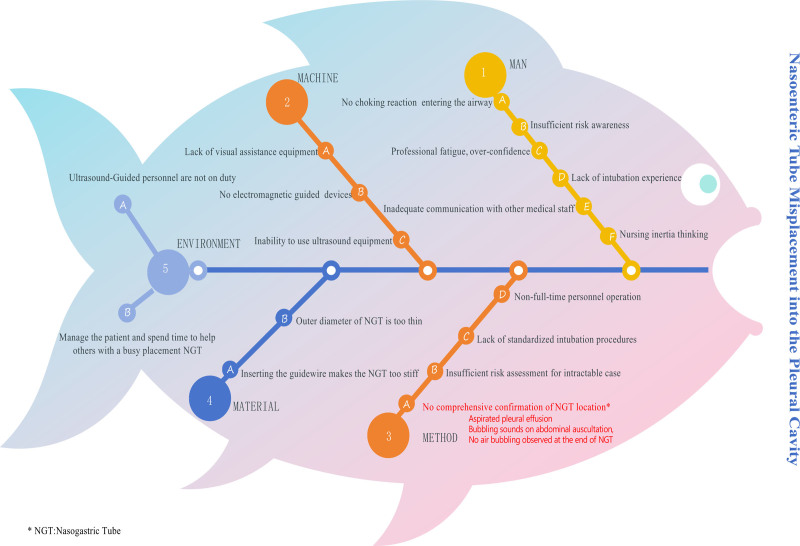
Fishbone diagram of RCA results. RCA = root cause analysis.

**Figure 3.**
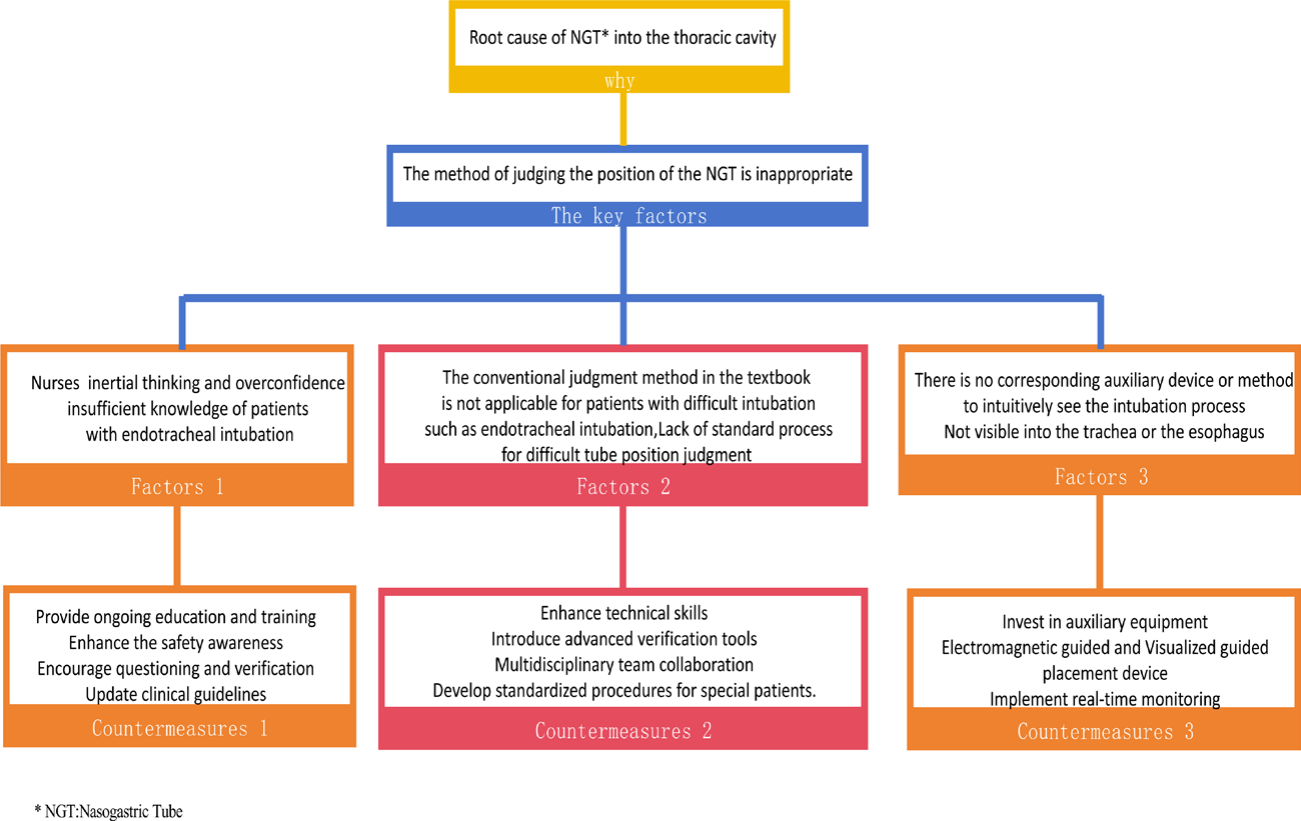
The key factors and countermeasures.

This case involved a patient with a tracheostomy who was on mechanical ventilation. Despite employing various verification methods during the tube placement process, the tube was still mistakenly inserted into the chest cavity, leading to a report to the Nursing Quality and Safety Management Committee. A joint investigation team with the Intensive Care Department was established to conduct in-depth interviews with the personnel involved in the tube placement to reconstruct the process. They used brainstorming to perform a root cause analysis from 5 aspects: man, machine, material, method, and environment. The results were presented in the form of a fishbone diagram as shown in Figure 2.Analyze key factors and formulate corresponding measures, as shown in Figure 3, to avoid the occurrence of similar events and ensure medical safety and patient safety.

The joint investigation team also discussed the anatomical and procedural factors. They highlighted that anatomically, the esophagus and trachea share a close pathway in the pharynx, which naturally increases the risk of a nasoenteric tube mistakenly entering the trachea. This risk is exacerbated in patients with tracheostomies who also have ICU-acquired weakness (ICU-AW), as their airway response to foreign bodies may be diminished, thereby reducing the cough reflex, an important safety signal. Moreover, among the procedural factors, the highly experience-dependent nature of blind insertion techniques suggests that less experienced nurses may struggle to accurately judge the tube’s position, especially when patients do not exhibit obvious discomfort symptoms, which increases the likelihood of misplacement.

## 6. Discussion

### 6.1. The incidence of pneumothorax

In 2021, Motta et al conducted an integrative review on nasogastric/nasoenteric tube-related adverse events, pointing out that respiratory complications are the most common, with pneumothorax being the most frequent issue. The incidence rate of the tube mistakenly entering the airway is as high as 3.2%. By calculating the data from 3 observational studies related to the complication of pneumothorax within this review, the incidence rates of pneumothorax are found to be 2.15‰ (9/4190), 4.94‰ (9/1822), and 6.76‰ (5/740) respectively.^[1]^These complications not only increase the risk of patient treatment but may also lead to extended hospital stays and increased medical costs, and can even result in patient death.

Traditional methods for confirming nasoenteric tube placement, such as aspiration, auscultation, and water bubble tests, have notable limitations.

### 6.2. Aspiration method limitations

This method involves drawing fluid from the tube to confirm placement. If gastric juice is aspirated, it is assumed the tube is in the stomach. However, this method may fail if the stomach is empty or has insufficient content. In this case, the nasoenteric tube was mistakenly inserted into the chest cavity of a patient with pleural effusion. The aspirated yellow fluid, resembling gastric juice, led to the incorrect assumption that the tube was in the stomach.

### 6.3. Auscultation method limitations

This method relies on listening for gurgling sounds when air is injected into the stomach. Interference from abdominal gas or bowel sounds can lead to misdiagnosis. In this case, the tip of the nasoenteric tube was submerged in the pleural effusion. By injecting air, pleural effusion created sounds similar to air passing through water, this sound, transmitted through the abdominal wall, which were misinterpreted as indicating the tube’s placement in the stomach. Furthermore, the auscultation method’s accuracy is highly dependent on the operator’s experience and auditory judgment, making it subjective and prone to errors, particularly in noisy environments or for less experienced operators^.[2]^

### 6.4. Water bubble test limitations

Submerging the tube’s end in water to detect air bubbles is a common method. If air bubbles emerge, it may have mistakenly entered the airway; if no air bubbles emerge, it is generally considered that the tube is in the stomach. However, no bubbles may be observed in cases of respiratory arrest or insufficient airflow, even if the tube is correctly positioned in the airway. Conversely, gas in the stomach can cause bubbles to form. In this case, the tube was in the thoracic cavity, where the presence of pleural effusion led to no bubbles being observed, resulting in misdiagnosis.

Traditional nasoenteric tube placement techniques and the post-placement tube localization techniques have challenges and limitations.

### 6.5. Blind placement technique deficiencies

The blind placement technique relies on the operator’s experience and the patient’s physiological responses, which may lead to inaccurate tube placement. The case involved a patient whose misplaced tube was not detected due to the loss of the cough reflex, X-ray examination is usually required to confirm the position of the intestinal tube, not only increasing medical costs and led to delayed feeding, but also exposing patients to radiation.^[1]^

### 6.6. Endoscopy and fluoroscopy-guided limitations

These methods improve accuracy but are technically challenging, expensive, and require transporting the patient to specific locations, adding to the patient’s transfer risks.^[3]^

### 6.7. Ultrasound-guided technique challenges

Ultrasound-guided placement reduces reliance on X-rays but requires high operator skill. It often necessitates collaboration between 2 professionals and is affected by factors such as pneumoperitoneum, intestinal distension, and obesity, which can impact operability.^[4]^

### 6.8. Post-placement tube localization techniques limitations

Advanced techniques like pH indication methods, carbon dioxide detection methods, and X-ray confirmation methods enhance accuracy. However, these inspection methods are conducted after the placement is completed, that is, after the occurrence of risk events, and such techniques cannot prevent the occurrence of complications. In this case, it was only after an X-ray examination that the issue was discovered.^[5]^

The advent of new technology has brought about innovations in the placement of nasoenteric tubes, reducing reliance on X-rays, minimizing patient radiation exposure, and decreasing the need for repeated placements, thus lowering pain and medical costs.

## 7. Electromagnetic guided placement technique

During placement, the localizer is positioned at the xiphoid-sternum junction. It creates a 3D coordinate system (X, Y, Z) with itself as the origin. When the nasoenteric tube with a guide wire enters this field, its path is tracked in real-time. The display terminal calculates and shows the guide wire’s tip trajectory as a 3D graphic, enabling real-time localization of the tube’s path.^[6]^

## 8. Visualized guided placement technique

This technology uses a miniaturized endoscope, allowing real-time observation of the patient’s gastrointestinal tract position on the screen and confirming its passage through the nasopharynx, esophagus, stomach, pylorus, and into the duodenum. The changes and trends in the gastrointestinal mucosa observed via visualization allow for more accurate determination of the tube’s position.^[7]^

## 9. Feasibility analysis of advanced technology implementation

In China, electromagnetic navigation and visualization navigation technologies have received strong support from national policies, especially in terms of training and cost-effectiveness. Medical and health institutions and professional academic groups under the National Health Commission have launched specialized training programs to help medical staff with traditional intubation experience to master these advanced technologies, which have been rapidly deployed against the backdrop of rapid information development. Although the initial investment is high, the National Healthcare Security Administration has effectively reduced the direct economic burden on patients and corporate costs through medical insurance coverage and centralized procurement strategies, while increasing the overall profits of enterprises. Moreover, these technologies can be selectively prioritized for patients with difficult intubation, and in the long run, the benefits brought by reducing intubation time, improving the success rate of intubation, reducing complications, enhancing patient satisfaction, and saving labor costs are significant. Therefore, despite limited resources, the implementation of these advanced technologies in China is feasible through policy support and cost-benefit optimization.

## 10. Summary and outlook

Traditional nasoenteric tube placement methods have limitations, especially in verifying path and location. Advances in medical technology highlight the need to enhance patient safety by integrating electromagnetic guided and visualized guided placement technique. Combining the 2 technologies could revolutionize nasoenteric tube placement. This will not only improve the efficiency and safety of clinical operations but also provide a more humane and comfortable therapeutic experience for patients. Future research and clinical practice should focus on leveraging these innovations to achieve better outcomes and patient satisfaction.

## Acknowledgments

We would like to express our gratitude to Professor Yuqun Chen for his guidance, Dongmei Wu and Qijie Li support. Special thanks go to the patient and their family for authorizing the report.

## Author contributions

**Data curation:** You Yuan, Huiming Gao, Yuanqin Qing.

**Formal analysis:** Xia Zhang, Junxi Chen.

**Funding acquisition:** You Yuan, Rujun Hu.

**Investigation:** You Yuan, Huiming Gao, Xia Zhang, Yuanqin Qing.

**Visualization:** Huiming Gao.

**Writing – original draft:** You Yuan.

**Writing – review & editing:** Wei Zhong, Rujun Hu.
